# PyF2F: a robust and simplified fluorophore-to-fluorophore distance measurement tool for Protein interactions from Imaging Complexes after Translocation experiments

**DOI:** 10.1093/nargab/lqae027

**Published:** 2024-03-12

**Authors:** Altair C Hernandez, Sebastian Ortiz, Laura I Betancur, Radovan Dojčilović, Andrea Picco, Marko Kaksonen, Baldo Oliva, Oriol Gallego

**Affiliations:** Department of Medicine and Life Sciences, Universitat Pompeu Fabra, Barcelona 08005, Catalonia, Spain; Department of Medicine and Life Sciences, Universitat Pompeu Fabra, Barcelona 08005, Catalonia, Spain; Department of Medicine and Life Sciences, Universitat Pompeu Fabra, Barcelona 08005, Catalonia, Spain; Department of Medicine and Life Sciences, Universitat Pompeu Fabra, Barcelona 08005, Catalonia, Spain; Department of Biochemistry, University of Geneva, 1205 Genève, Switzerland; Department of Biochemistry, University of Geneva, 1205 Genève, Switzerland; Department of Medicine and Life Sciences, Universitat Pompeu Fabra, Barcelona 08005, Catalonia, Spain; Department of Medicine and Life Sciences, Universitat Pompeu Fabra, Barcelona 08005, Catalonia, Spain

## Abstract

Structural knowledge of protein assemblies in their physiological environment is paramount to understand cellular functions at the molecular level. Protein interactions from Imaging Complexes after Translocation (PICT) is a live-cell imaging technique for the structural characterization of macromolecular assemblies in living cells. PICT relies on the measurement of the separation between labelled molecules using fluorescence microscopy and cell engineering. Unfortunately, the required computational tools to extract molecular distances involve a variety of sophisticated software programs that challenge reproducibility and limit their implementation to highly specialized researchers. Here we introduce PyF2F, a Python-based software that provides a workflow for measuring molecular distances from PICT data, with minimal user programming expertise. We used a published dataset to validate PyF2F’s performance.

## Introduction

Unravelling the structure of macromolecular complexes is necessary to understand molecular functions, interactions and dynamics that explain the mechanisms controlling the cell's biology. The structure of molecular assemblies can be solved at high resolution by a number of techniques such as X-ray crystallography, nuclear magnetic resonance (NMR) and cryo-electron microscopy. However, these methods are limited to solve only molecular assemblies that have been previously isolated from their physiological environment. This technical requirement prevents capturing functional conformations and structural dynamics that are central to the mechanism of cellular processes.

Fluorescence microscopy offers the unique opportunity to investigate the spatial organization of biological macromolecules within their physiological environment: the cell. Although light diffraction limits the resolution of fluorescence microscopy to 200–300 nm, different approaches have been developed that allow measurements at the molecular scale. Förster resonance energy transfer (FRET) is a microscopy technique that can estimate distances between fluorescent labels *in vivo*. However, FRET measurements are restrained to distances within the range of 2–10 nm, which limits the conformational space that can be characterized by this method. Localization microscopy is a technique that overcomes this restraint by estimating the centroid position of diffraction-limited fluorescence spots (1–3). With this method, protein complex subunits can be specifically labelled with fluorescent markers whose position can be determined at high precision (in the range of 20–30 nm) (2). Repetitive and reproducible measurement of the distance between two centroids allows for estimating their true separation with up to 1 nm precision regardless of the distance between the two fluorophores (1,4–10). Thus, the measurement of fluorophore-to-fluorophore distances can provide outstanding information about conformational changes and molecular interactions (6,8–10).

In practice, technical constraints limit the implementation of localization microscopy to interrogate protein complex structures *in situ*. Firstly, not all proteins can be observed as distinguishable diffraction-limited spots when labelled with a fluorophore in living cells. Secondly, the intrinsic dynamic nature of cellular processes prevents repetitive and reproducible imaging of the diffraction-limited spots. Thirdly, accurate measurements of distances between fluorophores requires the combination of sophisticated image pre-processing (i.e. subtraction of background noise, image registration and cell segmentation) and analysis (i.e. feature detection, Gaussian fitting and rejection of outliers) of the raw data.

Our group developed PICT (Protein interactions from Imaging Complexes after Translocation) (11,12), a live-cell imaging technique that enables (i) the inducible distribution of fluorescently labelled protein complexes in diffraction-limited spots and (ii) the repetitive and reproducible imaging to estimate distances with high precision. PICT employs yeast Sla2, a stiff rod-like protein that, in the absence of actin cables, clusters in flat and immobile platforms linked to the plasma membrane. PICT is based on the rapamycin-induced heterodimerization of FK506-binding protein (FKBP) and FKBP–rapamycin binding (FRB) domains (13). Upon addition of rapamycin, the protein complex harbouring a subunit labelled with FRB (bait–FRB) is recruited to Sla2 platforms or ‘anchor’ labelled with FKBP. The anchor and another subunit of the protein complex (prey) are labelled with distinguishable fluorophores [i.e. anchor–red fluorescent protein (RFP)–FKBP and prey–green fluorescent protein (GFP)]. Once the recruitment of the protein complex has succeeded, the centroid position for the anchor–RFP–FKBP and prey–GFP in the equatorial plane of the cell are determined and their separation is estimated. The spherical topology of yeast cells, combined with the stationary rod-shaped Sla2 anchor, allow repetitive and reproducible measurements, making PICT suitable to estimate the separation between fluorescent labels in the 2D image with a 2–5 nm precision (12). Moreover, PICT allows measuring the distance between the labelled subunit and the anchor–RFP–FKBP when the complex is recruited in different orientations simply by fusing the FRB tag to different subunits. Ultimately, integration of the distances measured in 2D images allows determination of the 3D coordinates of the fluorophores that label the complex subunits using the anchor–RFP–FKBP as a spatial reference point. This approach has been used to reconstruct the architecture of the exocyst and the conserved oligomeric Golgi (COG) complexes in living cells (12).

Unfortunately, the sample preparation, image pre-processing and analysis workflows necessary for a precise distance estimation with PICT still require specialized expertise and the installation and version compatibility control of multiple software programs such as FIJI/ImageJ (14), MATLAB (https://es.mathworks.com/) and R (https://www.r-project.org/). In practice, the broad implementation of the PICT method to resolve the architecture of protein assemblies suffers from optimal integration of multiple types of software and custom scripts, and the requirement for specialized skills in microscopy and image analysis that are rarely found in structural biology laboratories.

Here, we present PyF2F (Python-based Fluorophore-to-Fluorophore ruler), a Python-based open-source software for measuring distances between two fluorescent markers from diffraction-limited microscopy images obtained by PICT. PyF2F streamlines the analysis of PICT datasets and offers a robust platform for the measurement of fluorophore-to-fluorophore distances *in situ*. We improved the performance of yeast cell segmentation by taking advantage of pre-trained convolutional neural network (CNN) weights used in YeastSpotter (15). We updated the chromatic aberration correction approach to achieve subnanometre accuracy registration (6). We also incorporated the biophysical characterization of the anchor–RFP–FKBP to systematically monitor the quality and consistency of the different PICT datasets analysed. Overall, PyF2F is an accessible and robust tool that facilitates the reproducibility of fluorophore-to-fluorophore distance measurement in living cells. Together with the code and documentation, we provide a walkthrough tutorial that can be easily followed by users unfamiliar with this image analysis protocol. PyF2F can run standalone with a local installation or online using a Colab notebook (https://colab.research.google.com/drive/1kSOnZdwRb4xuznyQIpRNWUBBFKms91M8?usp=sharing).

## Materials and methods

### Software architecture

PyF2F has been implemented in Python 3.7 and runs standalone from any Linux or MacOS terminal. The standalone version has dependencies on the following packages:

- sci-kit image v. 0.19.2 (16).
- pymicro v. 0.5.1 (https://www.github.com/heprom/pymicro).
- vtk v. 9.1.0 (https://book.vtk.org/en/latest/index.html).
- trackpy v. 0.5.0 (https://doi.org/10.5281/zenodo.4682814).
- scipy v. 1.7.3 (17).
- numpy v. 1.21.2 (18).
- pandas v. 1.3.5 (https://pandas.pydata.org/).
- plotly v. 5.3.1 (https://plotly.com/).
- seaborn v. 0.12.2 (19).
- matplotlib v. 3.5.1 (20).
- h5py v. 2.10.0 (https://www.hdfgroup.org/solutions/hdf5/).
- keras v. 2.1.6 (https://keras.io/).
- lmfit v. 1.0.3 (21).
- opencv_python v.4.5.5.62 (https://opencv.org/).
- pillow v.9.5.0 (https://doi.org/10.5281/zenodo.10450403).
- tensorflow v.1.15.0 (https://doi.org/10.5281/zenodo.10126399).

PyF2F also integrates customized Python functions of the YeastSpotter (15) for an accurate yeast cell segmentation. YeastSpotter is a web-server tool that uses a pre-trained mask-RCNN model (22) re-trained on both brightfield and fluorescence microscopy images of *Saccharomyces cerevisiae* (15). For more information about the CNN architecture and training, see the work of Lu *et al.* (15).

### Workflow

PyF2F’s workflow consists of four major steps: (i) image registration; (ii) image pre-processing, spot detection, spot–pair linking and chromatic aberration correction; (iii) spot–pair selection; and (iv) outlier rejection and distance estimation. We provide recommended parameters given a system equipped with a ×100, 1.49 NA objective lens and a camera sensor with a pixel size of 6.45 μm. Although PyF2F can analyse datasets obtained by various imaging techniques (e.g. confocal, spinning disk or structured illumination microscopy), we recommend acquiring PICT datasets with wide-field fluorescence microscopy to minimize the loss of photons. The workflow and the expected outcome are illustrated with the distance estimation of a reference dataset (MKYSGA0048 yeast strain expressing the anchor–RFP–FKBP, the bait Exo70–FRB and the C-terminally tagged prey Sec5–GFP) (12) (see Supplementary Table S1). The reader can find the instructions to use PyF2F on our GitHub (https://github.com/GallegoLab/PyF2F).

#### Image registration

Before embarking on the fluorophore-to-fluorophore distance measurement, PyF2F calculates and corrects the chromatic aberration intrinsic to the employed microscope setup. Chromatic aberration is a geometrical distortion that occurs when using multiple-colour channel imaging, causing deviations between channels (23,24). To correct chromatic aberration, multi-colour fluorescent beads (TetraSpeck) are imaged and used as a reference to calculate the intrinsic chromatic aberration of the employed optical system. To achieve an accurate correction, it is critical to image a homogeneous distribution of beads throughout the camera sensor while leading to a negligible number of bead aggregates. Inspired by Niekamp *et al.* (6), we recommend combining a lower bead density [25–75 beads per field of view (FOV)] with the imaging of a grid of 10 × 10 FOV with an overlap of 90% between consecutive FOVs (6). This results in a uniform distribution of the fluorescent beads throughout the camera sensor with a minimal number of bead aggregates, which allows calculation of the chromatic aberration between channels with higher accuracy (Supplementary Figure S1).

**Figure 1.**
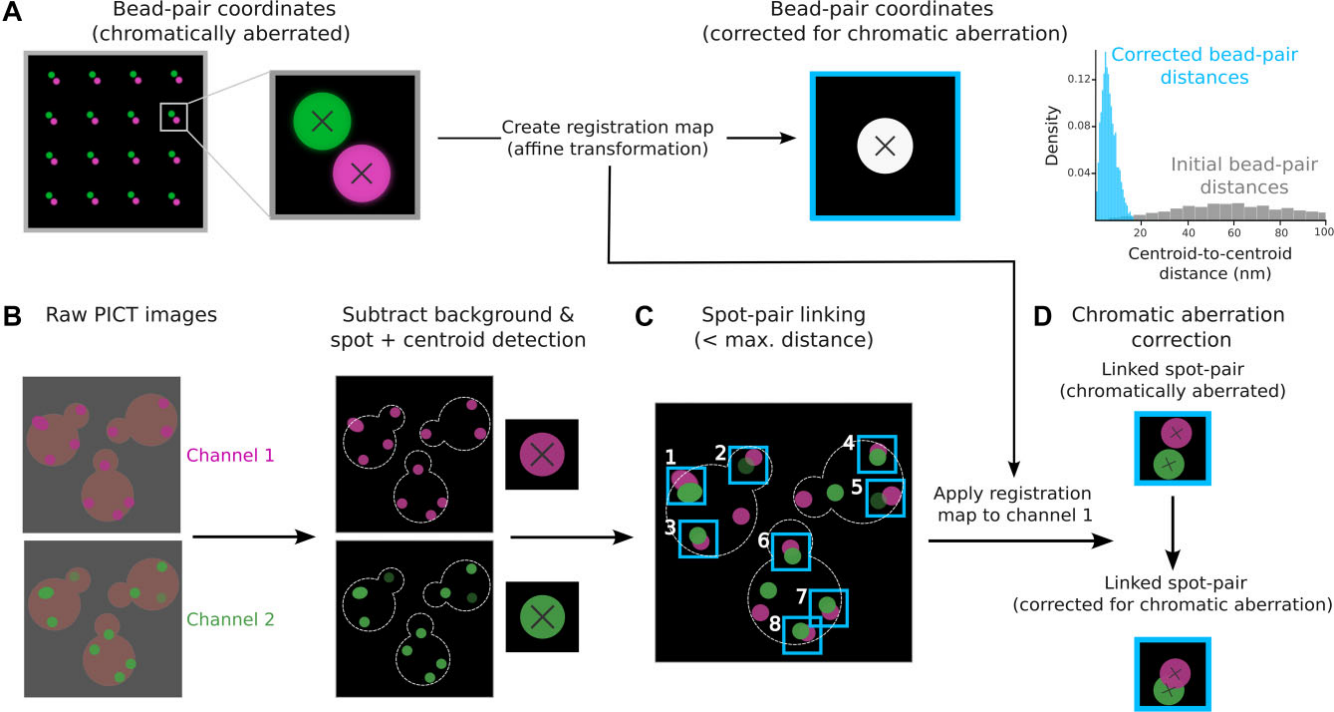
Scheme of image registration, image pre-processing, spot detection, spot–pair linking and chromatic aberration correction. (**A**) Image registration: a dataset of two-channel images of TetraSpeck beads (red fluorescence, channel 1-magenta; green fluorescence, channel 2-green) (left). The zoom-in illustrates the centroid coordinates of representative fluorescent beads (black cross) for each channel before (centre) and after (right) being aligned using an affine transformation. This allows creation of the registration map. As a control, the centroid-to-centroid Euclidean distances between two-channel bead coordinates are measured before (grey) and after (light-blue) the chromatic aberration correction (right). (**B**) Image pre-processing: two-channel images obtained in a PICT experiment are firstly pre-processed to remove the extracellular and cytoplasmic background fluorescence signal (left). Spot detection: the centroid coordinates of the fluorescent spots corresponding to the labelled anchor–RFP–FKBP (magenta) and prey (green) are localized (represented with a black cross in the zoom-in; centre). (**C**) Spot–pair linking: the most proximal spots detected in channel 1 and channel 2 and that are found within a maximum separation distance defined by the user are linked into ‘spot pairs’ (i.e. pairs of spots with ID 1–8, light-blue). (**D**) Chromatic aberration correction: finally, the centroid coordinates of detected spots in channel 1 are corrected for chromatic aberration using the registration map computed with the bead images (right).

The acquisition of images of beads generates a dataset of 100 two-channel images, which PyF2F splits into two datasets of 50 images: one dataset is used to calculate the registration map (representing the chromatic aberration between channels) and the other dataset is used to validate the registration accuracy (Supplementary Figure S1) (6). PyF2F utilizes *pymicro*’s point set registration function (*compute_affine_transform*) to calculate the registration map. The registration map is computed by applying an affine transformation, which corrects the beads’ coordinates of channel 1 (red fluorescence signal, which we represent as magenta to facilitate visualization) towards the beads’ coordinates of channel 2 (green fluorescence signal) used as reference (Figure 1A). PyF2F measures the accuracy of the image registration (target registration error, TRE) from the mean deviation of the beads’ centroid coordinates, in the *x-* and *y*-axis, between the two aligned channels (Supplementary Figure S1). We recommend proceeding with the subsequent image analysis steps only when the TRE is <1 nm.

#### Image pre-processing, spot detection, spot–pair linking and chromatic aberration correction

To initiate the analysis of PICT data, the background fluorescence of the images is corrected to localize the centroid of emission of fluorescent markers. Firstly, the extracellular background intensity is subtracted using the *rolling ball* algorithm (*sci-kit image*) with a rolling ball diameter slightly larger than the cell size. Secondly, to correct the uneven cytoplasmic fluorescence signal at the edge of the cell, PyF2F subtracts the median-filtered image from the background-subtracted image. The median-filtered image is computed using a diameter equal to twice the diffraction limit, which is sufficiently large to consistently erase the fluorescent spots from the image while preserving the cell contour (25) (Figure 1B). For the microscopy setup used as an example, we recommend using a rolling ball radius equal to 70 pixels and a median diameter of 11 pixels.

Subsequently, PyF2F performs the spot detection on the two channels independently using *trackpy*. Detected spots in channel 1 (anchor–RFP–FKBP) and channel 2 (prey–GFP) are then linked within a maximum separation distance between centroid coordinates set by the user (Figure 1C). For the microscopy setup used as an example, we recommend using a maximum separation distance of between 2 and 3 pixels for intra-assembly and inter-assembly distance measurements, respectively. Only linked spot pairs are selected for further analysis. Finally, the coordinates of selected spots in channel 1 are corrected for the chromatic aberration using the registration map saved previously (Figure 1D).

#### Spot–pair selection

At this step, PyF2F applies a sequence of filters to reject noisy centroid positions of detected spots to ensure that only reliable spot pairs are selected. Detected spot pairs are selected according to the following criteria.

**Figure 2.**
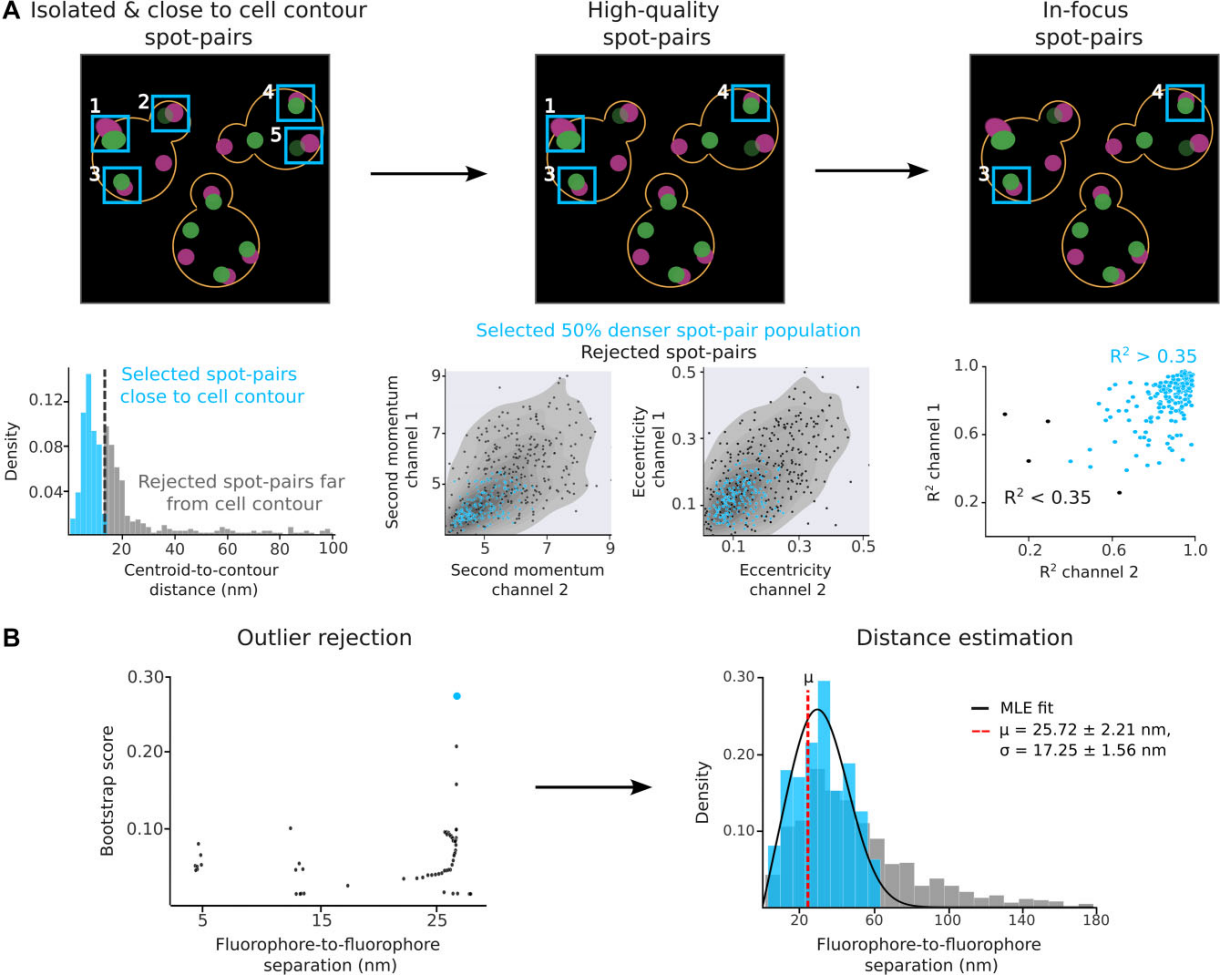
Scheme of spot–pair selection, outlier rejection and distance estimation for the reference dataset. (**A**) Spot–pair selection: the contour of the cell is approximated using the CNN weights of YeastSpotter (15). Isolated spot pairs are selected according to their separation from the closest neighbouring spot and the distance-to-cell contour (i.e. spot pairs with ID 1–5, light-blue, left). Only spot pairs with similar brightness (second momentum) and roundness (eccentricity) are selected (i.e. spot pairs with ID 1, 3 and 4, light-blue, centre). Lastly, only spot pairs whose intensity distribution in the *x*- and *y*-axis fits a Gaussian function are selected (i.e. spot pairs with ID 3 and 4, light-blue, right). (**B**) Outlier rejection and distance estimation: outliers are rejected using a bootstrap method (left, see Supplementary Notes S1 and S2) to determine the distance distribution without outliers that maximizes the likelihood for the estimated *μ* and *σ* (light-blue dot). The distance distribution is modelled by a Rician distribution (4,5) with an MLE to estimate the μ (red dashed line) and σ of the distribution (right). A representative PICT dataset of cells expressing anchor–RFP–FKBP, Exo70–FRB and C-terminally tagged Sec5–GFP was analysed to illustrate the main steps followed by PyF2F. Light-blue indicates selected spot pairs (A) and distance distribution without outliers (B).

- Isolated and close to cell contour events: PyF2F filters out overlapping spot pairs based on a maximum closest-neighbour distance (Figure 2A). We recommend using a maximum closest-neighbour distance equal to twice the diffraction limit minus one pixel (i.e. 10 pixels for the microscopy setup used as an example). Since PICT relies on the employment of plasma membrane-associated anchoring platforms, the centroid coordinates of fluorescent spots must be located at the edge of the cell equatorial plane (hereafter cell contour). PyF2F utilizes a neural network-based cell segmentation algorithm to draw the cell contour (15). Then, it selects only those spots's centroid coordinates encountered within a maximum distance to the cell contour (Figure 2A). We recommend using a maximum distance to the cell contour equivalent to twice the diffraction limit plus 2 pixels (i.e. 13 pixels for the microscopy setup used as an example).
- High-quality spot pairs: selection of high-quality spot pairs is done based on the highest dense group of spot pairs sharing similar properties in brightness (second momentum) and roundness (eccentricity) (Figure 2A). Spot pairs with a higher probability of being found are selected. We recommend using a cut-off of 0.5 to select the spot pairs that have a probability of ≥50% to be found (the 50% denser spot pair population).
- In-focus spot pairs: the intensity distribution in the *x*- and *y*-axis for each spot is evaluated with a 2D Gaussian fitting. PyF2F evaluates the quality of the interpolation with the goodness-of-fitting factor (*R²*) and selects spot pairs above the threshold set by the user (0–1) (Figure 2A). We recommend using a threshold of 0.35 to reject spot pairs with an inappropriate 2D-Gaussian intensity profile.

#### Outlier rejection and distance estimation

The last step estimates the true separation between the two fluorophore labels. The distribution of measured distances *d* that separates paired fluorescent spots follows a Rician distribution (4,5) defined as:


(1) \begin{equation*}p(d\, |\, \mu , \sigma ) = \left( {\frac{d}{{2\pi {{\sigma }^2}}}} \right) exp\left( { - \frac{{{{\mu }^2} + {{d}^2}}}{{2 {{\sigma }^2}}}} \right) {{I}_0}\left( {\frac{{d\mu }}{{{{\sigma }^2}}}} \right)\end{equation*}

where *μ* is the true separation between the fluorophores (the one we want to estimate), *σ* is the distribution variance and *I*_0_ is the modified Bessel function of integer order 0. The true separation *μ* can thus be computed with a Maximum Likelihood Estimate (MLE) (5). Due to the skewed nature of the distribution *d* (grey distance distribution, Figure 2B), outliers, especially if in the tail of the distribution, can fail the MLE. To reject these outlier spot pairs, we implemented a bootstrap method (Figure 2B; see Supplementary Note S1 and S2).

## Results

### Software validation

**Figure 3.**
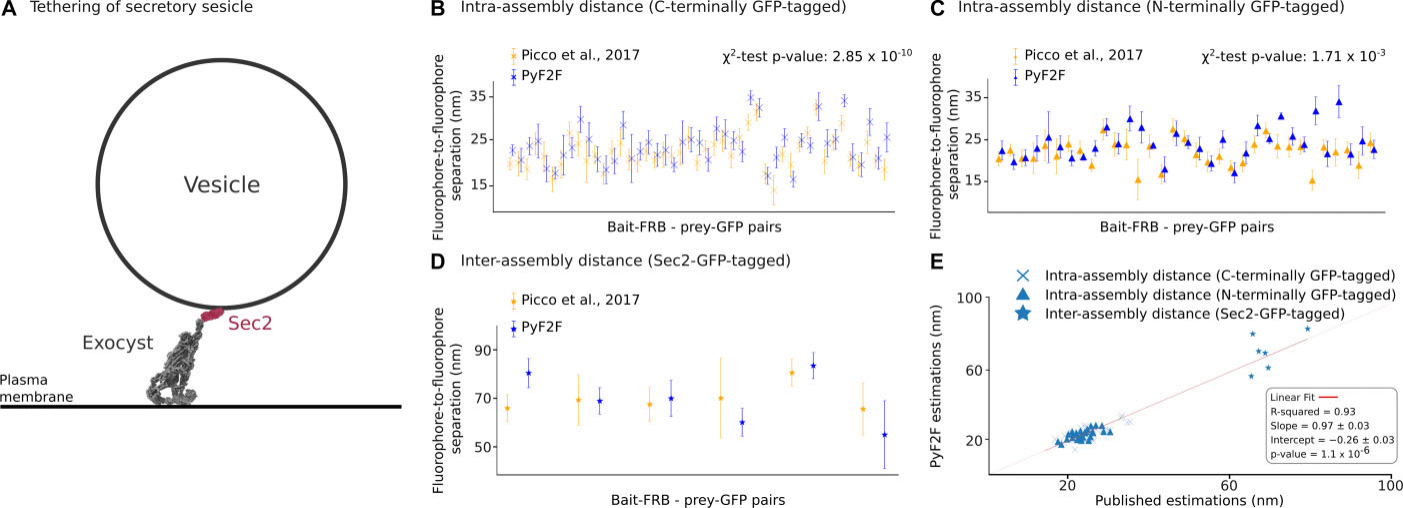
PyF2F validation. Validation of the PyF2F performance on a PICT reference dataset by comparing published distance estimations (orange, (12)) with the estimations obtained with PyF2F (blue) (see Supplementary Table S2). (**A**) The scheme illustrates a 2D section of the tethering of a secretory vesicle mediated by the interaction between the exocyst (grey, representation based on PDB 5YFP) (12) and Sec2 (red, cartoon representation using Biorender.com). (**B**) A total of 45 estimated distances between the anchor–RFP–FKBP and exocyst subunits tagged to GFP at their C-terminus. (**C**) A total of 33 estimated distances between the anchor–RFP–FKBP and exocyst subunits tagged to GFP at their N-terminus. (**B, C**) The difference between the set of distance estimations obtained by each approach was evaluated with a χ^2^ test. (**D**) Six distances between the anchor–RFP–FKBP and the GFP fused to the Sec2 C-terminus (inter-assembly distances). (**E**) The correlation between the published and the PyF2F distance estimations was evaluated with a linear fitting (*R*^2^ = 0.93; slope = 0.97 ± 0.03; intercept = −0.26 ± 0.03; *P*-value = 1.1 × 10^−6^).

To evaluate the PyF2F performance, we employed a reference PICT dataset that had been used to reconstruct the architecture of the exocyst complex bound to a secretory vesicle (12) (Supplementary Table S2). The exocyst is a conserved hetero-octameric complex (composed of Sec3, Sec5, Sec6, Sec8, Sec10, Sec15, Exo70 and Exo84) that tethers secretory vesicles to the plasma membrane in exocytosis. Exocyst function relies on the binding of its Sec15 subunit to Sec2, a guanyl-nucleotide exchange factor that is associated with the vesicle surface (26) (Figure 3A). The dataset included images of cells where the exocyst had been recruited to anchor–RFP–FKBP platforms in eight different orientations (i.e. the FRB tag had been fused to eight different exocyst subunits). Including all the orientations, we compared a total of 78 intra-assembly distance estimations: (i) 45 distances between the anchor–RFP–FKBP and the GFP tag fused to the C-terminus of exocyst subunits (Figure 3B) and (ii) 33 distances between the anchor–RFP–FKBP and the GFP tag fused to the N-terminus of exocyst subunits (Figure 3C). We also compared six inter-assembly distance estimations (distances between the anchor–RFP–FKBP and the GFP fused to the Sec2 C-terminus) (Figure 3D; Supplementary Table S2).

First, we used a χ^2^ test to corroborate that the set of measurements obtained by PyF2F reproduces the published set of distances. We excluded the distance measurements for Sec2–GFP because the available number of measurements in the reference dataset was not large enough (*n* = 6) to perform a reliable χ^2^ test. The χ^2^ test showed no significant differences between the set of distance estimations (*P*-value of 2.85 × 10^–10^ and 1.7 × 10^–3^ for the C- and N-terminal intra-assembly distance measurements, respectively) (Figure 3B, C). We also performed a linear fitting to assess the correlation between the distance estimations of the two approaches. The strong correlation (*R*^2^ = 0.93, *P*-value = 1.1 × 10^–6^), the estimated slope (0.97 ± 0.03) and the intercept (–0.26 ± 0.03) suggest that the results obtained with PyF2F are coherent and reproducible with respect to the distances measured in Picco *et al.* (Figure 3E). Overall, the coefficient of determination *R*^2^ of the linear regression and the χ^2^ test indicate that PyF2F delivers reliable distance estimations from PICT datasets.

### PyF2F to systematically assess PICT datasets

A fundamental assumption underlying *in situ* distance measurement with PyF2F is that the PICT assay grants reproducible and repetitive imaging of the bait–FRB/prey–GFP assembly, necessary for estimating the RFP-to-GFP distance with nanometer precision. However, reliable measurements require: (i) a robust microscopy setup ensuring unvarying imaging and (ii) the consistent *in vivo* performance of the anchor–RFP–FKBP platforms. The construction of the anchor–RFP–FKBP platforms depends on the intrinsic ability of Sla2 clusters to assemble on the yeast plasma membrane and the stabilization of these platforms facilitated by latrunculin A (LatA)-induced actin depolymerization. Unfortunately, small deviations from optimal conditions, on both the imaging setup and the cellular construction of anchoring platforms, are likely to impact on the data quality and the subsequent distance measurements. To strengthen the robustness of the approach, PyF2F features a measurable criterion to infer the consistency of the anchor–RFP–FKBP imaging and to supervise if the PICT assay has been performed optimally. PyF2F utilizes a quantitative biophysical analysis to compare PICT datasets (Supplementary Figure S2). PyF2F measures the second momentum of the anchor–RFP–FKBP spots to monitor changes in their structure and dynamism (see Supplementary Note S3). PyF2F measures the intensity (brightness) of the anchor–RFP–FKBP spots as a proxy of the Sla2 copy number that populates anchoring platforms. If the anchor–RFP–FKBP imaging and properties are conserved among different PICT datasets, the correlation between the mean second momentum and the mean intensity will be preserved. Supplementary Note S3 and Supplementary Figure S2 present an illustrative experiment and the expected output when assessing the reproducibility of optimal and artefactual PICT experiments.

## Discussion

PyF2F is a Python-based software that requires minimal user programming expertise to estimate the average separation between two fluorescently labelled proteins imaged by PICT. PyF2F overcomes the limitations of existing approaches by providing a unified application for the structural analysis of protein complexes translocated to engineered anchoring platforms (with a precision of 2–5 nm). Compared with the available software (12), improvements in the image registration (6), yeast cell segmentation (15) and quality control of the anchor–RFP–FKBP make PyF2F a portable and robust tool. We provide the required tutorials to facilitate the use of PyF2F by other laboratories (https://colab.research.google.com/drive/1kSOnZdwRb4xuznyQIpRNWUBBFKms91M8?usp=sharing).

The fact that the existing computational approach ran over custom and obsolete versions of several software programs (ImageJ plugins, MATLAB and R) complicates its installation (incompatible R/RStudio versions) and usage (requires skills on programming and image analysis). In addition, the former approach relied on poorly controlled parameters (i.e. PICT sample preparation, image registration and cell segmentation) that challenged the reproducibility of the measurements obtained by non-expert users (12). PyF2F is easy to install and to use, and it features various capabilities that enhance the robustness of the analysis. Indeed, PyF2F is designed to be employed by a broader community of users that do not necessarily combine expertise in light microscopy, image analysis and structural biology. PyF2F modularity allows the user to tune the analysis according to the particularities of each dataset (see https://github.com/GallegoLab/PyF2F). We here discuss the main features and technical considerations for an optimal employment of PyF2F.

To improve the image registration used in the former approach, we applied a workflow that achieves dense and homogenous bead imaging throughout the entire camera sensor while minimizing bead aggregates (6). To provide the user with objective measurements of the registration quality, the PyF2F further calculates the TRE (6) (see illustrative comparison in Supplementary Figure S3). Remarkably, although reference bead datasets are imaged on the same support and culture medium as the cells, the bead imaging does not recapitulate the intracellular milieu or the exact axial separation between the equatorial anchor–RFP–FKBP and the glass surface. Thus, one must notice that small deviations are intrinsic to bead-corrected PICT datasets. Although the direction of the residual aberration is not isotropic, the relative position of the spots is random in a large FOV. As such, small errors in the chromatic aberration will have a random contribution to the distance estimates that will result in a larger *σ* of the distance estimate. Further recommendations to minimize the registration error are given in Supplementary Note S4. Supplementary Figure S4 illustrates the outcome of a dataset analysis without chromatic aberration correction.

PyF2F leverages the arrangement of diffraction-limited spots with respect to the yeast plasma membrane to specifically select spot pairs that (i) result from the recruitment of protein assemblies to Sla2 anchoring platforms and that (ii) had been imaged at the equatorial plane of the cell (25). The former approach used a cell segmentation based on the pixel intensity that poorly estimated the cell boundaries, especially when the cytoplasmic fluorescence was not evenly distributed or cells clustered together (25) (Supplementary Figure S5). PyF2F takes advantage of the deep learning tool YeastSpotter to calculate the cell contour with higher accuracy (15), minimizing false negatives (loss of spot pairs adjacent to regions of the plasma membrane that had not been properly segmented) and false positives (spurious spot pairs that contaminate the dataset) (see Supplementary Figure S5 and Supplementary Note S2).

PyF2F measures the separation between fluorescent proteins labelling the subunits of an assembly and the anchor–RFP–FKBP, where the assembly is recruited. Consequently, the anchor–RFP–FKBP also serves as a spatial reference to integrate multiple distance measurements and to reconstruct the architecture of protein assemblies directly in living cells (12). Data integration hinges on the assumption that different measurements are obtained under comparable experimental conditions, such as imaging settings and anchoring performance, but how to monitor the validity of such premise had not been considered previously. With PyF2F, non-specialized users are equipped to assess, in an unbiased manner, the coherence between datasets, a crucial step to derive structural and mechanistic models from data integration (see Supplementary Note S3 and Supplementary Figure S2).

PyF2F streamlines the *in situ* intermolecular distance estimation. In combination with the PICT technique, the software generalizes the structural measurements within and between protein complexes directly in living cells. Overall, PyF2F contributes with unique capabilities to the flourishing of cellular structural biology. Together with other light microscopy techniques, *in situ* cross-linking mass spectrometry, cryo-electron tomography and integrative modelling, PyF2F and PICT narrow the gap towards the ultimate goal of resolving molecular structures *in vivo*.

## Supplementary Material

lqae027_Supplemental_File

## Data Availability

The code and data underlying this article are available on GitHub at https://github.com/GallegoLab/PyF2F under the MIT licence. Permanent DOI: PyF2F (https://doi.org/10.5281/zenodo.8014795).

## Notes

Present address: Radovan Dojčilović, Vinča Institute of Nuclear Sciences, National Institute of the Republic of Serbia, University of Belgrade, PO Box 522, 11001 Belgrade, Serbia.
