# Supplementary material for: PyF2F: a robust and simplified fluorophore-to-fluorophore distance measurement tool for Protein interactions from Imaging Complexes after Translocation experiments
Source: NAR Genom Bioinform. 2024 Mar 12;6(1):lqae027. doi: 10.1093/nargab/lqae027 (PMC10939353; doi:10.1093/nargab/lqae027)
Supplement: lqae027_Supplemental_File [file lqae027_supplemental_file.pdf]

PyF2F: A robust and simplified fluorophore-to-fluorophore distance measurement tool for Protein interactions from Imaging Complexes after Translocation experiments

Supplementary Material

Altair C. Hernandez[1], Sebastian Ortiz[1], Laura I. Betancur[1], Radovan Dojčilović[1,2], Andrea Picco[3], Marko Kaksonen[3], Baldo Oliva[1], and Oriol Gallego[1]

[1] Department of Medicine and Life Sciences, Universitat Pompeu Fabra, Barcelona 08005, Catalonia, Spain. [2] Present address: Vinča Institute of Nuclear Sciences, National Institute of the Republic of Serbia, University of Belgrade, P.O. Box 522, 11001 Belgrade, Serbia. [3] Department of Biochemistry, University of Geneva, 1205 Genève, Switzerland

Note S1. Bootstrap method for the outlier rejection

Due to the skewed nature of the distance distribution, precise distance estimation requires rejection of possible outliers. We performed the outlier rejection by using the following bootstrap method (for more details, see *Methods* section from Picco A. et al., 2017 (**1**):

- Each datapoint (x_i) from the dataset X is rejected, one at the time, and for each rejection we compute the log-likelihood given an initial estimate of μ and σ:

$$\log L_i(\mu, \sigma) = \sum_{x \in X} \log p(x; \mu, \sigma) \qquad \text{(log-likelihood)}$$

  The datapoint that is less likely to belong to the dataset is the one whose rejection gives the worst log-likelihood: $\min_{x_i} \{\log L_i(\mu, \sigma)\}$. This datapoint is rejected and a new estimate of μ and σ is computed.

- The process is iterated by exploring a given percentage of the dataset defined by the user, starting from the largest distances (which are those defining the tail of the distribution, where outliers, if present, are more problematic), has been sampled for rejection. We do not expect to reject as many data points, but ⅓ of the dataset is a safe parameter to ensure that we had enough sampling.

- Two subsequent rejections *i* and *i+1* will give two estimates of μ. Their difference, $\delta\mu_i = \mu_{i+1} - \mu_i$, will decrease when most outliers are rejected and the score will thus be maximal:

$$p_{\delta\mu_i} = \frac{1/\delta\mu_i}{\sum_j 1/\delta\mu_j},\ p_{\delta\sigma_i} = \frac{1/\delta\sigma_i}{\sum_j 1/\delta\sigma_j} \quad \text{(scores for each iteration)}$$

The μ, σ and the ensemble of data points that are retained after all these iterations are those that maximise a scoring function defined as:

$$S(p_{\delta\mu_i}, p_{\delta\sigma_i}) = -p_{\delta\mu_i}\log\left(p_{\delta\mu_i}\right) - p_{\delta\sigma_i}\log\left(p_{\delta\sigma_i}\right) \quad \text{(scoring function)}$$

The total score $S(p_{\delta\mu_i}, p_{\delta\sigma_i})$ will be maximal when both scores $p_{\delta\mu_i}$ and $p_{\delta\sigma_i}$ will be similarly maximised ($\max_i (S(p_{\delta\mu_i}, p_{\delta\sigma_i}))$).

Note S2. Interpreting the bootstrap method for the outlier rejection

The bootstrap method aims to find the distance distribution that maximises the log-likelihood for estimating the μ and σ parameters given a Rice distribution (**2**). We can achieve this by iteratively rejecting the data points that are less likely to belong to the dataset (false positives - outliers) and consequently minimise the log-likelihood. As shown in Figure 2B, the bootstrap method scores the estimation of μ of the resulting distribution after an outlier rejection (black dots), giving us a notion about the likelihood to find outliers in that distribution. The lower the score, the more likely to find outliers in that distribution (**Figure 2B**). Although this approach is suitable for detecting outliers (the one that maximised the scoring function), it faces some limitations that need to be considered when interpreting the results:

1) Two parameter estimation: two parameters (μ and σ) are estimated at the same time throughout a minimisation process (see **Note S1**) using the maximum likelihood estimation. When convergence is not achieved equally for μ and σ (due to the presence of large number of outliers, or to a low number of observations within the distance distribution) one may find two or more "peaks" (maximum relative scores) for μ and σ estimations.

2) Dataset quality: contamination of the dataset (number of outliers) is directly influenced by the quality of the images analysed and the number of the anchor-RFP-FKBP - prey-GFP paired spots that are suitable for the distance measurement (number of true positives). We assumed that the contamination of the dataset with false positives should not be higher than 20% of the data points. Larger contaminations due to poor-quality images, such as the presence of

out-of-focus spots, inefficient anchoring, inaccurate cell segmentation, etc., might lower the performance of the bootstrap method for obtaining accurate estimations.

Note S3. A practical case to illustrate PICT assay assessment with PyF2F

As a proof-of-concept, we analysed two PICT datasets from the same yeast strain (i.e. OGY1322 cells expressing the anchor-RFP-FKBP, Exo70-FRB as bait and Sec5-mNeonGreen (Sec5-mNG), being mNG a green fluorescent protein equivalent to the 3xGFP for the current example). Each PICT dataset had been acquired by treating cell samples with different batches of latrunculin A (LatA): a fresh batch (batch 1) and a diluted batch that had been stocked as solution for more than 6 months (batch 2). LatA is employed to inhibit the polymerization of the actin cables, thereby facilitating the static anchoring of Sla2-based platforms on the plasma membrane (**1**). Diluted LatA might lead to partial actin polymerization that will impact on the properties of the anchor-RFP-FKBP platforms (structure and dynamism). As shown in **Figure S2A**, the fluorescent spots corresponding to the anchor-RFP-FKBP imaged in different biological replicates can be grouped in the 2D space defined by the mean logarithmic intensity and the mean second momentum. Although the datasets acquired in cells treated with different LatA batches do not show a significantly different second momentum on average, the statistical dispersion of their distribution suggests that the underlying structure or dynamism of the anchor-RFP-FKBP platforms is different. Indeed, the mean logarithmic intensity of the anchor-RFP-FKBP spots is significantly higher for the PICT dataset treated with the batch 1 of LatA than the dataset obtained with the diluted batch 2 (batch 2), suggesting that subtle differences in the LatA batch might impact on the structure of the anchor-RFP-FKBP platform (i.e, the copy number that populates the anchor-RFP-FKBP platforms).

The consequences that deficient anchor-RFP-FKBP platforms might have on the distance estimations is likely to depend on many factors, such as the nature of intra-assembly or inter-assembly interactions. It is probably the latest the more sensitive to slight defects in the PICT assay due to its intrinsic transient behaviour. We have further compared the estimations for the distance (μ) and standard deviation (σ) for the same yeast strain (i.e. cells expressing anchor-RFP-FKBP, Exo70-FRB as bait and Sec5-mNG as prey) in four biological replicates for each LatA batch (**Figure S2B**). No significant differences could be observed between estimations throughout replicates imaged with the different LatA batches (**Figure S2B).** However, estimations of σ in cells treated with the LatA batch1 are comparably smaller than the σ estimations done in cells treated with the LatA batch 2.

Note S4. Imaging recommendation

Several factors must be carefully monitored by the users to minimise the registration error when aligning the two-channel PICT images: change of focus during the image acquisition, temperature variation, mechanical drift, changes in the light path, sample dynamics, etc (**3, 4, 5**). To minimise the likelihood of aberrations we suggest: 1) controlling the axial drift with an automated focus correction system (we use the perfect focus system (PSF), Nikon), 2) adding a delay between the movement to a new FOV and the image acquisition to minimise X-Y mechanical drift (we use a 2.5 seconds waiting-time), 3) using an objective with apochromatic optical lenses, 4) imaging the fiducial markers and the PICT sample on the same holder, support and media, and 5) using a dual-band bandpass filter cube that does not require any filter change along the whole experiment to minimise light path variations.

Figure S1. Accuracy of image registration

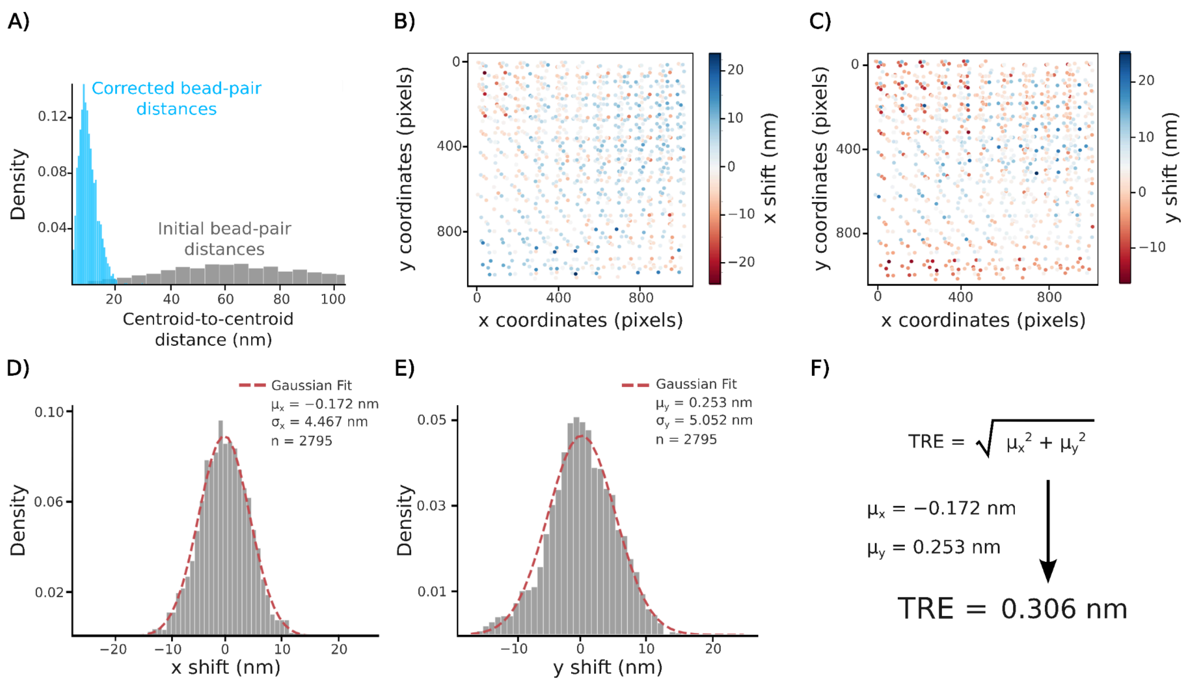

A) The distribution of centroid-to-centroid distances between the beads coordinates in the two channels is depicted before (grey) and after (cyan) two-channel alignment. **B, C**) After alignment, we can assess the shift distribution (deviation of bead coordinates in one channel with respect to the other) along **B**) the *x*-axis and **C**) the *y*-axis through the field of view. Each dot shows a single bead colour-coded to indicate the coordinate shift between the two channels of the same bead. **D, E**) The average deviation (μ_x^2 and μ_y^2) between the beads' *x* and *y* positions in the two channels after alignment can be derived by fitting each lateral distribution to a Gaussian function. **F**) The registration accuracy (target registration error, TRE) is given by the following equation:

$$TRE = \sqrt{\mu_x^2 + \mu_y^2}$$

We proceed to the subsequent steps of the image analysis only when the registration accuracy is less than 1 nanometer.

Figure S2. Monitoring the performance of the anchor-RFP-FKBP

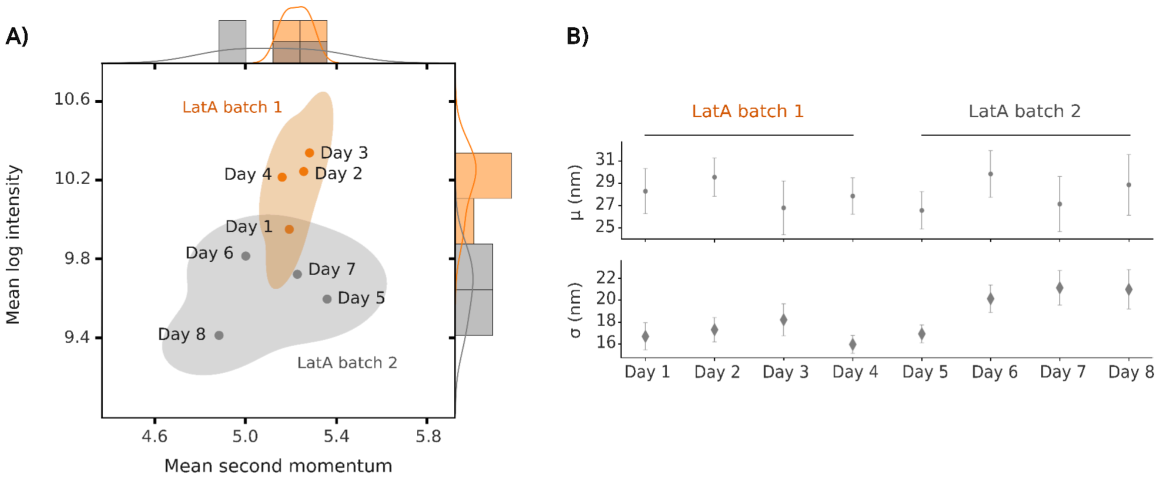

Comparative biophysical characterization of the anchor-RFP-FKBP platforms (channel 1) in PICT experiments performed with different batches of LatA. Cells expressing the anchor-RFP-FKBP, bait: Exo70-FRB, and prey: Sec5-mNG, were imaged on different days (LatA batch 1: days 1-4; LatA batch 2: days 5-8). **A)** Detected anchor-RFP-FKBP are evaluated using the mean second momentum (*x*-axis) and the mean log intensity (*y*-axis). **B)** The estimated fluorophore-to-fluorophore separation (µ) and standard deviation (σ) are compared for the biological replicates of cells treated with each batch.

Figure S3. Image registration used in the former approach

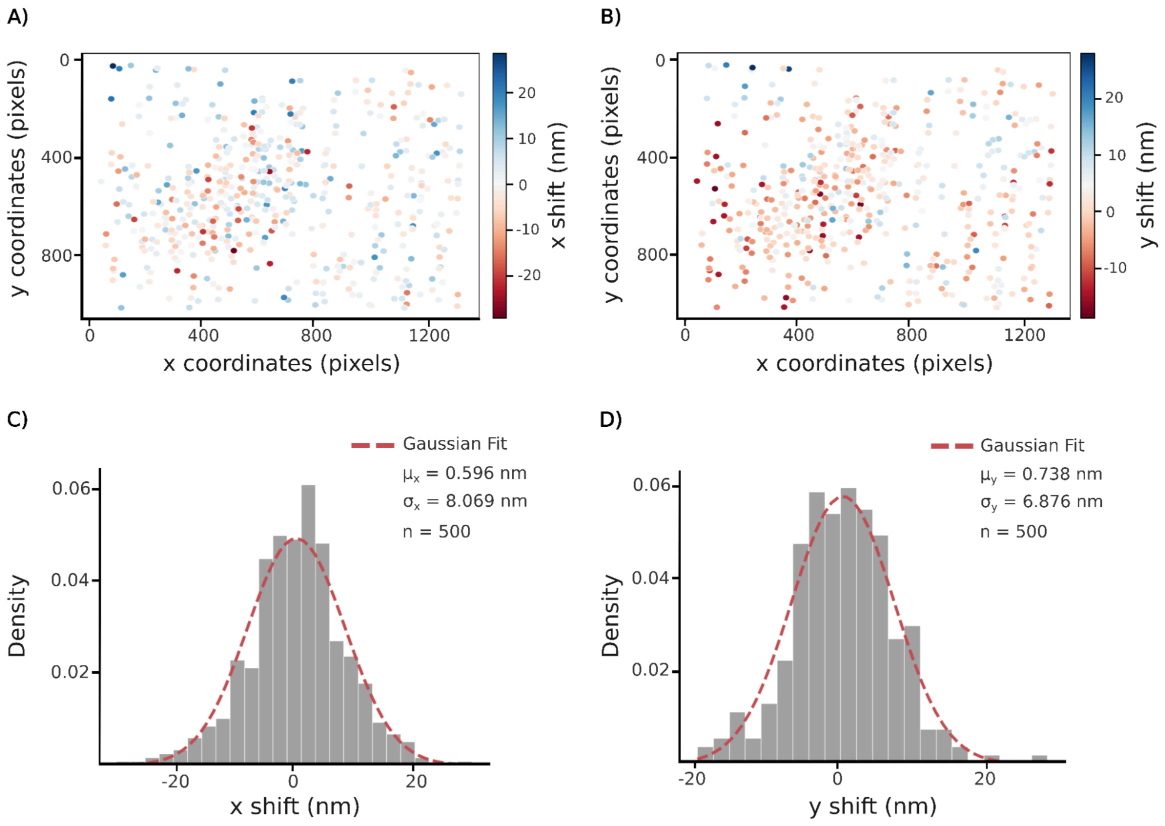

Illustration of the chromatic aberration correction performance used in Picco et al., 2017. **A, B**) The imaging of reference beads resulted in an inhomogeneous distribution of beads over the camera sensor (white areas without beads). After alignment, we can assess the shift distribution (deviation of bead coordinates in one channel with respect to the other) along **A)** the *x*-axis and **B**) the *y*-axis through the field of view. **C, D**) The average deviation (μ_x^2 and μ_y^2) between the beads' *x* and *y* positions in the two channels after alignment can be derived by fitting each lateral distribution to a Gaussian function. Note that the detected beads (n < 1000) are not able to recapitulate the chromatic aberration over the entire system (**4**).

Figure S4. Distance estimate without chromatic aberration correction

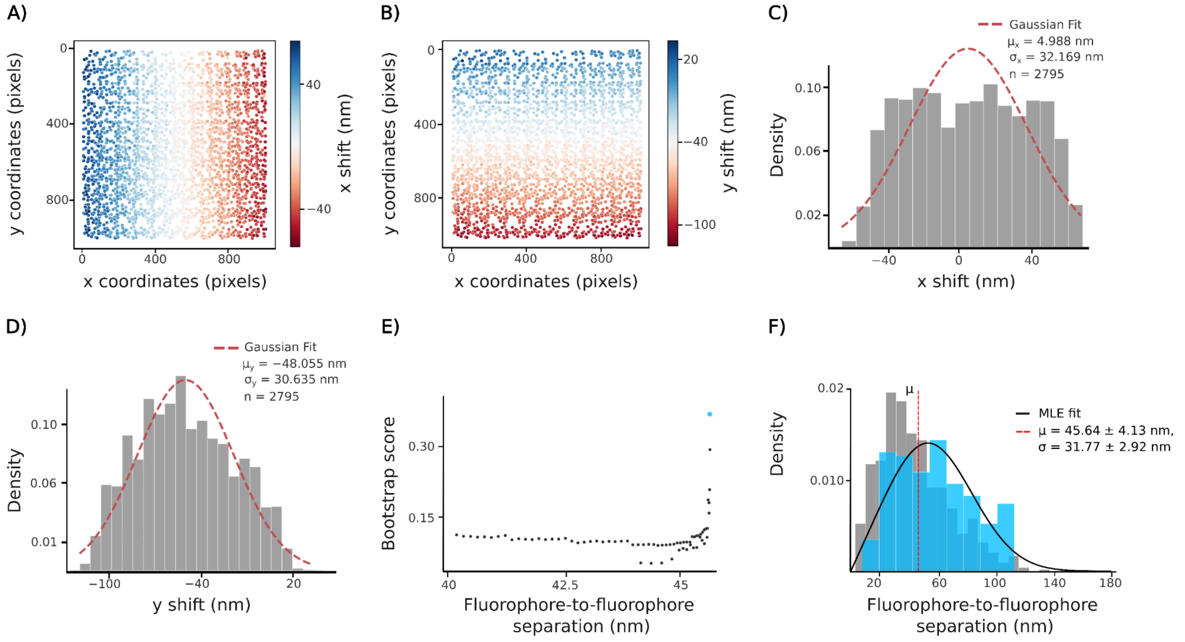

A case example of the distance estimate obtained without applying chromatic aberration correction. **A, B)** The same beads illustrated in Figure S1 are not corrected for the chromatic aberration of the system. The omission of the chromatic aberration correction is reflected as a large shift in **A**) the *x*-axis and **B**) the *y*-axis. **C, D)** Consequently, the average deviation μ_x^2 **C**) and μ_y^2 **D**) between the beads' *x* and *y* positions in the two channels is larger, and their distribution shows a wider standard deviation (σ) when fitted to a Gaussian function. **E, F)** The omission of image registration and chromatic aberration correction steps leads to an inaccurate outlier rejection **E**) and a large distance estimation with a distance distribution more skewed towards the right tail **F**).

Figure S5. Comparison of the yeast cell segmentation

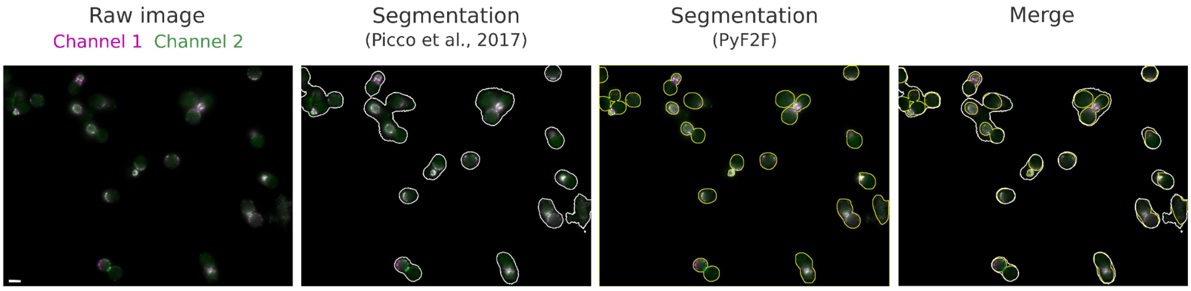

We used a raw image from a PICT dataset (left column) to compare the cell segmentation performance of the former approach (Picco et al., 2017, second column) and PyF2F (third column). In the previous segmentation approach (second column), which was based on pixel intensity, segmented cell contour (grey) did not capture the cells boundary accurately (Picco et al., 2017). PyF2F cell segmentation (third column) takes advantage of YeastSpotter deep learning tool to segment the yeast cells with high accuracy (yellow). Scale bar: 2 µm.

Table S1. Yeast strains

MKYSGA0048	*MATa, can1Δ::STE2pr-LEU2, lyp1Δ::, his3Δ1, leu2Δ0, ura3Δ0, LYS2+, tor1-1, fpr1Δ::klURA, SLA2-RFP-FKBP::natNT2, EXO70-FRB::hphNT1, SEC5-3xmyeGFP::kanMX4.*
OGY1322	*MATα, can1Δ::STE2pr-LEU2, Lyp1Δ::, his3Δ1, leu2Δ0, ura3Δ0, LYS2+, tor1-1, fpr1Δ::klUra, SLA2-RFP-FKBP::natNT2, EXO70-FRB::hphNT1, SEC5-mNG::kanMX4.*

Table S2. Comparison of distance estimates

The performance of PyF2F was assessed using a reference PICT dataset (**1**) by comparing the results obtained with PyF2F (**μ**_PyF2F – **SE$_{μ}$**_PyF2F) with the published estimates (**μ**_Picco – **SE$_{μ}$**_Picco). We compared 45 distance estimates corresponding to the separation between the anchor-RFP-FKBP and C-terminally-tagged exocyst subunits (GFP_C); 33 distance estimations corresponding to the separation between the anchor-RFP-FKBP and N-terminally-tagged exocyst subunits (GFP_N); and 6 inter-assembly distance estimates corresponding to the separation between the anchor-RFP-FKBP and Sec2 fused to GFP (Sec2_GFP_C).

μ: Distance estimation.
SE$_{μ}$: Standard error of the distance estimation.

Bait	Prey	μ_Picco (nm)	SE$_{μ}$_Picco (nm)	μ_PyF2F (nm)	SE$_{μ}$_PyF2F (nm)
Sec3-FRB	Sec5_GFP_C	19.92	1.37	23.03	1.21
Sec3-FRB	Sec6_GFP_C	20	2.73	20.82	2.64
Sec3-FRB	Sec8_GFP_C	18.72	2.4	23.98	1.97
Sec3-FRB	Sec10_GFP_C	24.52	2.47	25.09	3.84
Sec3-FRB	Sec15_GFP_C	21.64	3.28	18.8	2.95
Sec3-FRB	Exo70_GFP_C	16.41	2.79	17.8	1.34
Sec3-FRB	Exo84_GFP_C	19.79	3.07	21.89	4.54
Sec6-FRB	Sec3_GFP_C	26.96	2.48	23.62	2.31
Sec6-FRB	Sec5_GFP_C	24.49	2.76	30.02	2.96

Sec6-FRB	Sec15_GFP_C	20.55	4.88	25.51	3.83
Sec8-FRB	Exo70_GFP_C	22.04	2.02	20.98	2.8
Sec8-FRB	Exo84_GFP_C	17.61	2.35	18.59	3.15
Sec6-FRB	Exo70_GFP_C	22.71	2.49	20.57	2.9
Sec6-FRB	Exo84_GFP_C	24.67	3.02	28.75	3.12
Sec8-FRB	Sec3_GFP_C	20.9	2.36	21.15	5.46
Sec8-FRB	Sec5_GFP_C	20.15	1.98	22.72	1.97
Sec8-FRB	Sec6_GFP_C	22.87	2.24	24.82	2.69
Sec8-FRB	Sec10_GFP_C	20.68	1.73	21.97	2.62
Sec15-FRB	Sec3_GFP_C	21.99	2.86	23.06	3.42
Sec15-FRB	Sec5_GFP_C	21.84	2.01	19.68	1.57
Sec10-FRB	Sec3_GFP_C	22.5	2.79	24.86	4.79
Sec10-FRB	Sec5_GFP_C	25.85	2.51	25.43	2.78
Sec10-FRB	Sec8_GFP_C	20.17	1.71	24.73	2.77
Sec10-FRB	Sec15_GFP_C	23.25	1.88	20.79	2.56
Sec10-FRB	Exo70_GFP_C	24.29	2.43	27.93	2.63
Sec10-FRB	Exo84_GFP_C	25.14	2.41	26.67	3.26
Sec15-FRB	Sec6_GFP_C	21.75	2.73	25.24	2.04
Exo70-FRB	Sec6_GFP_C	25.87	1.98	22.69	2.3
Exo70-FRB	Sec8_GFP_C	29.3	1.74	34.96	1.58
Exo70-FRB	Sec10_GFP_C	31.69	1.94	32.66	2.08
Sec15-FRB	Sec8_GFP_C	17.72	2.24	17.21	1.94
Sec15-FRB	Sec10_GFP_C	13.91	3.35	21.37	2.35
Sec15-FRB	Exo70_GFP_C	22.12	1.72	25.91	2
Sec15-FRB	Exo84_GFP_C	19.68	2.48	16.42	1.82
Exo70-FRB	Sec3_GFP_C	24.02	1.3	25.35	1.45
Exo70-FRB	Sec5_GFP_C	26.88	1.36	24.12	1.77
Exo70-FRB	Sec15_GFP_C	32.73	1.73	32.92	3.14
Exo84-FRB	Sec15_GFP_C	20.49	2.77	24.59	3.1
Exo84-FRB	Exo70_GFP_C	24.78	1.76	25.48	2.22

Exo70-FRB	Exo84_GFP_C	29.09	1.77	34.25	1.38
Exo84-FRB	Sec3_GFP_C	21.04	1.72	21.47	3.07
Exo84-FRB	Sec5_GFP_C	19.5	2.31	19.73	2.71
Exo84-FRB	Sec6_GFP_C	24.79	2.57	29.45	3.04
Exo84-FRB	Sec8_GFP_C	20.6	1.53	21.19	2.49
Exo84-FRB	Sec10_GFP_C	18.6	2.36	25.91	3.31
Exo70-FRB	Sec5_GFP_N	20.46	1.67	22.42	2.27
Sec3-FRB	Sec5_GFP_N	22.51	1.41	19.73	1.79
Sec3-FRB	Sec6_GFP_N	20.56	2.06	20.72	1.86
Sec3-FRB	Sec10_GFP_N	20.56	3.65	22.95	3.03
Sec3-FRB	Sec15_GFP_N	23.65	3.61	25.64	6.05
Sec6-FRB	Sec3_GFP_N	21.09	4.19	23.31	2.63
Sec8-FRB	Sec3_GFP_N	24	2.02	20.66	1.82
Sec8-FRB	Sec5_GFP_N	22.47	2.26	20.91	0.83
Sec8-FRB	Sec6_GFP_N	18.92	1.44	22.94	1.83
Sec8-FRB	Sec10_GFP_N	27.37	2.51	28.02	2.05
Sec15-FRB	Sec3_GFP_N	23.91	2.83	24.06	2.45
Sec15-FRB	Sec5_GFP_N	23.83	3.63	30.03	2.97
Sec10-FRB	Sec5_GFP_N	15.55	4.82	27.95	3.73
Sec10-FRB	Sec6_GFP_N	23.48	2.72	23.72	0.32
Sec10-FRB	Sec15_GFP_N	16.78	2.21	17.98	2.97
Sec15-FRB	Sec6_GFP_N	27.51	2.63	26.5	2.93
Exo70-FRB	Sec6_GFP_N	25.29	1.81	24.39	1.5
Sec15-FRB	Sec10_GFP_N	21.47	2.83	22.92	2.7
Exo70-FRB	Sec3_GFP_N	19.72	2.36	19.35	1.81
Exo70-FRB	Sec15_GFP_N	21.05	1.8	25.13	2.09
Exo70-FRB	Exo84_GFP_N	18.38	1.63	17.16	2.38
Exo84-FRB	Sec3_GFP_N	19.45	2.11	21.86	2.62
Exo84-FRB	Sec5_GFP_N	23.92	1.95	28.39	2.49
Exo84-FRB	Sec6_GFP_N	27.15	1.77	25.26	1.12

Exo84-FRB	Sec10_GFP_N	23.47	2.74	30.64	0.78
Sec3-FRB	Exo70_GFP_N	23.28	2.32	25.82	2.13
Sec8-FRB	Exo84_GFP_N	23.34	1.74	23.89	1.78
Sec10-FRB	Exo84_GFP_N	15.36	2.37	31.89	3.3
Sec8-FRB	Exo70_GFP_N	23.26	1.8	21.66	3.02
Sec10-FRB	Exo70_GFP_N	22.04	3.21	34.08	3.83
Sec3-FRB	Exo84_GFP_N	22.55	2.01	21.58	2.48
Exo84-FRB	Exo70_GFP_N	18.92	3.25	24.71	3.36
Sec15-FRB	Exo84_GFP_N	24.32	2.23	22.71	2.19
Sec3-FRB	Sec2_GFP_C	65.22	5.13	78.73	5.65
Sec6-FRB	Sec2_GFP_C	68.31	9.69	68	5.1
Sec10-FRB	Sec2_GFP_C	66.69	6.51	68.96	7.05
Sec15-FRB	Sec2_GFP_C	69.06	15.41	59.76	5.33
Exo70-FRB	Sec2_GFP_C	78.8	5.18	81.57	5.03
Exo84-FRB	Sec2_GFP_C	64.87	10.03	55	13.07

REFERENCES

1. Picco,A., Irastorza-Azcarate,I., Specht,T., Böke,D., Pazos,I., Rivier-Cordey,A.-S., Devos,D.P., Kaksonen,M. and Gallego,O. (2017) The in vivo architecture of the exocyst provides structural basis for exocytosis. *Cell*, **168**, 400–412.

2. Churchman,L.S., Flyvbjerg,H. and Spudich,J.A. (2006) A non-Gaussian distribution quantifies distances measured with fluorescence localization techniques. *Biophys. J.*, **90**, 668–671.

3. Mortensen,K.I., Sung,J., Flyvbjerg,H. and Spudich,J.A. (2015) Optimized measurements of separations and angles between intra-molecular fluorescent markers. *Nat. Commun.*, **6**, 8621.

4. Niekamp,S., Sung,J., Huynh,W., Bhabha,G., Vale,R.D. and Stuurman,N. (2019) Nanometer-accuracy distance measurements between fluorophores at the single-molecule level. *Proc. Natl. Acad. Sci.*, **116**, 4275–4284.

5. Lopez,A.C., Conesa Mingo,P., Oña Blanco,A.M., Gomez-Pedrero,J.A. and Sorzano,C. (2023) Real-Time Correction of Chromatic Aberration in Optical Fluorescence Microscopy. *Methods Appl. Fluoresc.*
